# A Literature Review Informing an Operational Guideline for Inertial Sensor Propulsion Measurement in Wheelchair Court Sports

**DOI:** 10.3390/sports6020034

**Published:** 2018-04-13

**Authors:** Jonathan B. Shepherd, Daniel A. James, Hugo G. Espinosa, David V. Thiel, David D. Rowlands

**Affiliations:** 1 School of Engineering and Built Environment, Griffith University, Brisbane QLD 4111, Australia; j.shepherd@griffith.edu.au (J.B.S.); d.thiel@griffith.edu.au (D.V.T.); d.rowlands@griffith.edu.au (D.D.R.); 2 Centre of Excellence for Applied Sport Science Research, Queensland Academy of Sport, Queensland Sport and Athletics Centre, Brisbane QLD 4111, Australia; 3 SABEL Labs, Brisbane QLD 4111, Australia; dan@qsportstechnology.com

**Keywords:** inertial sensors, wheelchair sport, wheelchair propulsion, performance, IMU

## Abstract

With the increasing rise of professionalism in sport, teams and coaches are looking to technology to monitor performance in both games and training to find a competitive advantage. Wheelchair court sports (wheelchair rugby, wheelchair tennis, and wheelchair basketball) are no exception, and the use of microelectromechanical systems (MEMS)-based inertial measurement unit (IMU) within this domain is one innovation researchers have employed to monitor aspects of performance. A systematic literature review was conducted which, after the exclusion criteria was applied, comprised of 16 records. These records highlighted the efficacy of IMUs in terms of device validity and accuracy. IMUs are ubiquitous, low-cost, and non-invasive. The implementation in terms of algorithms and hardware choices was evidenced as a barrier to widespread adoption. This paper, through the information collected from the systematic review, proposes a set of implementation guidelines for using IMUs for wheelchair data capture. These guidelines, through the use of flow-charts and data tables, will aid researchers in reducing the barriers to IMU implementation for propulsion assessment.

## 1. Introduction

It has been suggested that 15% of the world’s population live with a disability and 2.2% of the population are functionally limited to a significant degree [1]. At elite levels in wheelchair court sports, a steep increase in participation has occurred over the last decade [2]. This is attributed to not only a large increase in awareness but also to advances in modern wheelchair technology [3] and an increased professionalism across wheelchair sports. Winning margins are becoming smaller as performance density increases. For instance, a delay of 1.73 s was the difference in the women’s 1500 m T54 wheelchair race between gold and bronze medal in Athens, 2004, whereas in London, 2012, this gap had shrunk to 0.61 s [2]. Subsequently, athletes and supporting staff are investing a considerable amount of time and resources to achieve best performance [4] with technological development to aid skill acquisition being one offshoot of this. 

**Figure 1:**
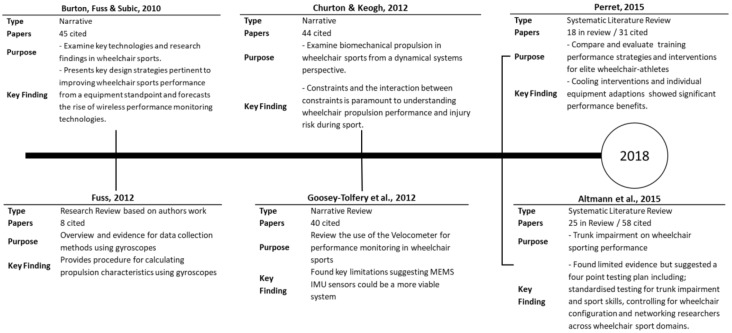
Profile of literature reviews pertinent to the measurement of wheelchair propulsion.

Although literature pertaining to wheelchair sport has been reported as sparse [2], there have been literature reviews pertinent to the measurement of wheelchair propulsion (Figure 1).

Burton et al. [3] primarily focused on the equipment and mechanics of wheelchair technologies and encompasses a section on performance measurement concluding how future development of monitoring equipment will raise performance levels. They categorized propulsive testing into two conditions: laboratory-controlled either static roll testing comprising of continuous belts or rolling rollers, or dynamic roll testing primarily tested in a performance environment. The measurement technology used in the dynamic performance assessment included static video cameras with image analytics software, global positioning system (GPS) sensor units, speedometers, inertial measurement units (IMUs), strain gauges, and load cells [3].

Researchers gravitated towards the use of on-board measurement technologies with a focus on speedometer type devices and IMUs. The reluctance to use GPS is primarily due to wheelchair court sports being played in GPS-denied environments. Goosey-Tolfery et al. [5] reviewed the use of a velocity meter (termed a velocometer) for propulsion assessment, which was referred to as a speedometer in the Burton et al. review [3]. This technology can be either analogue in nature, for example ticker tape timers, or more sophisticated digital measurements, for example with the aid of optical encoders. They concluded that there are key limitations to this technology; the system needs to be manually calibrated after each participant, the weight of 0.7 kg could affect maneuverability, transmission equipment that is attached to the chair might not pass for competition use due to sporting regulations, and the system requires post-processing, therefore concurrent feedback is not possible. Goosey-Tolfrey et al. (2012) suggested that IMU systems could eliminate many of the velocometers’ limitations, commenting that they are a more viable system for future use. Fuss [6] performed a research review into the use of gyroscopes, one sensor found in an IMU, using both experiential and empirically evidenced knowledge accrued over the seven years prior. He concluded that gyroscopes were a valid way of accurately ascertaining dynamic performance metrics.

Compared to other technologies used for wheelchair court sports, IMUs have the advantages of size, weight, integration, cost, ease of implementation, ubiquitous nature, and low power consumption, although they can be prone to significant instrument biases and drifting due to noise and device instability [7]. Furthermore, they can be packaged with other hardware allowing telemetry which affords real-time feedback [3].

One key advantage of microelectromechanical systems (MEMS) IMU technologies is that they can be used in situ. Practically, this allows wheelchair athletes to be assessed in their standard performance environment, either in game or training scenarios which ensures representative learning design. Representative learning design is a term coined by Pinder et al. (2011) that encompasses designing “dynamic interventions that consider interacting constraints on movement behaviours”. The term encapsulates the classical Brunswikian philosophy of task representative design as well as requiring these methodological principles to be adopted in all learning and performance environments. Mann et al. [8] suggests that representative learning design is critical for “ensuring that critical perception-action links remain intact” and the process is “fundamental to generality of experimental results” [9].

Churton and Keogh [10] reviewed hand rim wheelchair propulsive performance during wheelchair sports from a constraints-led approach of dynamical systems theory. Task, organism, and environmental constraints were all found to play an important role in understanding performance and the suggestion was to consider the synergism and interaction of all the constraints to increase propulsive performance and to reduce injury. Thus, measurement technology must be flexible enough to measure organism, environmental, and task constraints. 

Altmann et al. [11] and Perret et al. [2] performed recent systematic literature reviews focusing on trunk impairment and performance intervention strategies respectively. Both evidenced limited empirical research in the field of wheelchair performance, however commented that the development of technologies to measure performance parameters would aid the optimisation of performance [2,11]. The parameters of propulsion performance described in the context of wheelchair rugby but generalisable to other wheelchair court sports, were described by Chua et al. [12].

**Figure 2:**
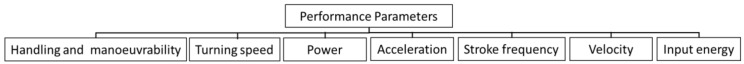
Schematic of the key performance parameters of wheelchair court sports as outlined by Chua et al. (2010).

Field testing provides a feasible way to get an indication of the performance parameters (Figure 2), and if monitored periodically allows for strength and weaknesses to be highlighted [4] and with appropriate training, to be incrementally improved. The use of IMUs to perform performance-based measurement in both game and training settings has been regarded as “highly feasible” [13]. Although the use of IMU technology is both cost-effective and user-friendly, the reliability of the measurements is highly dependent on the processing algorithms [14]. The creation and use of these processing algorithms, and how to implement these sensors for relevant performance feature extraction is a barrier to the wider adoption of IMUs for performance analysis in wheelchair sport. 

Simple IMU performance analysis, based on the identification of strokes in terms of numbers, amplitude, length, and periodicity, is reliant on the activity being cyclic with little fluctuation in frequency [12]. Typically, wheelchair court sports exhibit a range of different activity patterns with perturbations in speed, acceleration, and push frequencies common in game play. Practically, this means algorithms that are being used for game or multiple skill monitoring must not be reliant on static thresholds detecting peaks in IMU data, rather employing more sophisticated algorithms for performance assessment. As there is empirical evidence that wheelchair propulsion characteristics can be monitored using IMUs, this review will systematically assess the instances that accelerometers, gyroscopes, and magnetometers have been used for performance analysis in wheelchair court sport. This paper disseminates information on how the data is analysed in terms of feature extraction (Table 1), the analytic methodologies (Table 2) used to manipulate the data, and the physical instrumentation implementation (Table 3) in terms of hardware and sensor placement.

## 2. Methods

A review was conducted (current as of 10 January 2018) searching six scholarly databases (Google Scholar, Scopus, Web of Science, Proquest, ScienceDirect, and Sage Journals) The scholarly journals with the relevant search information is documented (Table 4).

**Figure 3:**
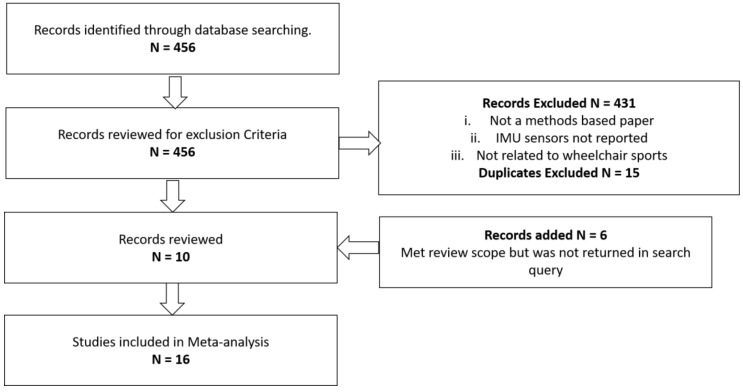
Schematic of the meta-analysis process. IMU, inertial measurement unit.

The inclusion criteria required for a manuscript to be accepted are that: it must be a methods-based research article from a scholarly journal, contain the use of inertial sensors (either gyroscopes, accelerometers, magnetometers, or a combination), and be applied in a wheelchair sports setting. To ensure reliability of the meta-analysis, two authors independently screened the results, returning the same outcome. A total of 456 records were returned on the search. From this a total of 431 records did not meet the inclusion criteria, a further 15 duplicate records were removed, with the remaining 10 articles included. Six articles that were not returned directly from the search, but met the entry criteria were added to the review increasing the total review to sixteen articles. These articles were all found through references from the included texts. A schematic of the literature review methodological processes is presented (Figure 3).

The information extracted from the included papers addressed questions including: (i) when, where, and by whom the research was published; (ii) what performance parameters were being measured; (iii) what algorithms were used to extract these performance parameters; (iv) which IMU hardware was implemented and the specifications of this hardware; and (v) what validation procedures were used to ensure measurement accuracy and validity. 

## 3. Results

Key features from all relevant records included the geographical spread, unique publication avenues, and the discipline spread. In total, the records appeared across nine unique journal publications. The journals spanned differing disciplines including biomechanics (*n* = 3), clinical rehabilitation and medicine (*n* = 3), and engineering and technology (*n* = 10). The geographical spread was also diverse with publications from researchers in North America (*n* = 1), Australasia (*n* = 7), and Europe (*n* = 8).

A clear trend emerged with all papers reporting inertial sensors to assess propulsive elements. Consequently, a comparison between these records could be made in terms of feature extraction (Table 1), the analytic methodologies used to extract these performance features (Table 2), and the hardware instrumentation implementation (Table 3). The only paper that was not compared in the tables was that of Starr et al. [15]. The paper utilised the commercially available MVN BIOMECH link motion capture system (Xsens, Enschede, The Netherlands) to assess shoulder biomechanical differences during propulsion between novice and expert uses. As it uses this commercial system, the paper gives limited information in the way data is collected and analysed, rather focusing on the shoulder kinematics attained from the motion capture software. 

To extract these features, software algorithms must be used; Table 2 below highlights the variety of algorithmic processes that were utilised. 

Another consideration of the review was to document the hardware these studies implemented (Table 3). Details including the number of IMU sensors being used, the sampling rate, if there was real-time communication and how this was achieved, and where the sensors were positioned were extracted. 

Common practice when implementing novel hardware or software measurement solutions is to undergo a validation process to ensure the accuracy and repeatability of measurements. The validation technology used to correlate the measurement outcome for the papers was also outputted from the meta-analysis (Table 5).

## 4. Discussion

The use of inertial sensors in wheelchair court sports is widespread and multinational with research from Australia, England, the Netherlands, Italy, and the United States of America. The publication avenues are also diverse with nine (60%) different journals featuring in the study. The primary discipline of these journals being technology-and-engineering-based (64%), although medical (22%) and biomechanics (14%) journals also provided publication avenues for inertial sensor work in wheelchair sports. The wide geographical and multidisciplinary spread evidences the global importance of this research. It additionally underpins that researchers within this domain must look outside their own disciplines to gain a full understanding of the use of inertial sensors in wheelchair sports. 

All the records (16/16) that were included in the review investigated propulsive elements, highlighting the importance of propulsion in wheelchair court sport performance. This is in line with previous reporting in the literature. Cavedon et al. [26] found that speed and endurance tasks correlated with game related statistics concluding the importance of propulsion in game scoring. To support this, qualitative interviews from nine elite wheelchair court sports athletes [27] indicated that propulsion was a principle element of performance.

Subcategorising the elements of propulsion, as shown in Table 1, it is evident that IMUs have been used to detect and classify a wide range of propulsive elements. The most popular element that was investigated was ground speed with 10/15 (66%) reporting this. The most prevalent way that the researchers measured this principle component was from the angular velocity attained from the rate gyroscope sensors that were rigidly mounted on the wheelchair wheels. This method was employed by 8/9 (89%) of the records that measured ground speed with Bergamini et al. [18] being the only record not employing this strategy. Instead, the authors used a threshold-based peak finding method from accelerometer data which was rigidly mounted to the chair, to ascertain speed. However, this method is only accurate in isolation as a purely linear propulsion task. 

Furthering the use of wheel-mounted gyroscopes for propulsion analysis, Chua et al. [13] found high feasibility in using gyroscopic sensors for measuring wheelchair kinematic data allowing for the calculation of distance the wheel travelled, if the radius of the wheel is known. The implemented algorithm requires an accurate wheel radius measurement, which varies with tire pressure and deformation due to player loading. Therefore, to ensure measurement accuracy for tire radius measurements, a standardised approach, for example an adaption of Moore et al. [28] wheel radius measurement protocol, is recommended. 

To ascertain accurate measurement of wheelchair speed rather than wheel speed a common frame of reference should be defined. One way to achieve this is through the measurement of wheel camber and a subsequent computational adjustment to align the wheel sensors into the same local frame of reference as the wheelchair as detailed in Pansiot et al. [16]. Five out of nine authors who used gyroscopes to find angular velocity reported performing a computational adjustment due to camber. Shepherd et al. [24] provided a different algorithmic approach to this using an open sourced Attitude and Heading Reference System (AHRS) orientation filter [29] to ensure sensor alignment by placing the sensors in a global frame of reference. The advantage of this method is the algorithm is camber agnostic and computationally inexpensive. 

The calculation of trajectory and positioning was also shown to be viable using inertial sensors with 7/15 (46%) of articles reporting positional tracking. Positional measurements provide insights that allow for performance analysis, but are also important for detecting and improving match strategy [3]. To ascertain positional information from inertial sensors, complex algorithmic processing and sensor fusion is required. A total of 33% (5/15) of the records used only IMUs for tracking (Pansiot et al. [16]; van der Slikke et al. [14,20,30]; [24]). Xu et al. [7] used a local reference GPS with an Extended Kalman Filter, and Usma-Alvarez et al. [21] used manually synced video and timing data to enable positional information.

When implementing IMUs for in-game scenarios, specific propulsive factors need to be accounted for algorithmically. An example of this is wheel skidding, which regularly occurs in wheelchair court sports. Van der Slikke et al. [14] proposed a correction algorithm that combines a rate gyroscope with an accelerometer in a complementary filter, calculating a weighted correction factor, allowing for the adjustment of potential skidding moments. Chua et al. [12] commented that push frequencies and acceleration patterns fluctuate with game play, therefore rendering static threshold based peak crossing detection algorithms inappropriate for in-game monitoring. 

The review uncovered that filtering data was a common algorithmic strategy to smooth the outputted data with 11/15 (73%) records reporting a type of data filtering. The most common type of filtering employed was noise filtering, allowing for feature extraction. Bergamini et al. [18] used a zero lag 4th order Butterworth filter with a 12 Hz cut off frequency with a further 4 Hz low pass filter for the forward component. Mason et al. [19] used a 2nd order low pass Butterworth filter with a 20 Hz cut off frequency. Chua et al. [12,13] used a 2nd order Savitzky–Golay filter and encompassed a window width of 25 samples. Other researchers used advanced filtering to fuse different IMU sensors data allowing for state estimation to mitigate against errors. Van der Slikke et al. [14,20,23,25,30] used a complementary filter to weigh the accelerometer and gyroscope, Xu et al. [7] used an extended Kalman filter to combine 5 Hz GPS data as a reference point to the rotation data from the gyroscope, and Shepherd et al. [24] used Madgwick’s [29] AHRS IMU orientation filter. 

As evidenced by Table 3, a wide variety of implementations were used in terms of number of sensors, sensor ranges, sampling frequencies, real-time capabilities and the mounting positions. Ten out of 15 (66%) of records utilised multiple IMUs and 6/15 (40%) reported the use of both accelerometers and gyroscopes, with only Shepherd et al. [24] reporting the use of a magnetometer, although the authors decided not to use this in their calculations due to a reported ferromagnetic disturbance. The accelerometer range varied from ±2 to ±168 g, the rate gyroscope ranged from +1200 to +6000°/s, and sampling frequencies between 30 Hz and 256 Hz were reported. As reported by Mason et al. [19], even with an unattainable speed of 6 m/s, the angular velocity of the sensor would be +1161°/s and therefore all of the authors used gyroscopes that are theoretically suitable. To further evidence the suitability of rate gyroscope measurement, Hiremath et al. [22] used +6000°/s, which could theoretically accurately measure 17.778 m/s on a 24” wheel. Low sampling frequencies were reported to be a source of error, with Mason et al. [19] commenting that the “magnitude and stability of the sampling frequency, which at approximately 50 Hz, may have been inadequate to determine rapid changes”. The mean sampling rate used was 130 Hz with both the median and mode equating to 100 Hz. 

Real time IMU data transmission was also proven successful which was evident in 3/15 (20%) studies. Two of the records used radio frequency antennas and reported indoor ranges between 20 m and 30 m, and the third, Hiremath et al. [22], used short-range Bluetooth and reported a small data transmission loss of 0.3%. Sensors where mounted in a variety of places with the wheel being the most common position followed by a rigidly mounted chair sensor. Wrist sensor (Bergamini et al. [18]) and full upper body sensor [15] positions were used to provide more detailed kinematic performance elements in propulsion. 

A variety of studies used different methods to validate the reliability of IMUs for propulsion performance analysis [31,32,33]. Mason et al. [19] investigated the reliability and validity of inertial sensors on wheelchair court sports in comparison to high speed video. In terms of speed, the sensor was found to be reliable, never exceeding a coefficient of variation of 0.9% at any speed. Peak speed was also proven valid using an IMU device with a coefficient of variation of 1.6%. Hiremath et al. [22] developed a gyroscope-based wheel monitoring system that estimates speed and distance and provides real time feedback in a laboratory setting. They found percentage errors from distance, speed and angular velocities were less than 3% for all trials.

Pansiot et al. [16] utilised wheel-mounted IMU units for providing real-time velocity, heading, ground distance covered, and motion trajectory. For linear distance, Pansiot et al. [16] validated the IMU output against a laser distance range finder which gave an uncalibrated average error of 53 mm over a 9 m trial. Furthermore, Pansiot et al. [16] validated the IMU outputs against known ground markings for positional tracking finding an average error of 94 ± 94 mm or a 0.76% distance error over a figure-8 loop. The use of a laser range finder and known ground markings was also evidenced by Shepherd [24].

Van der Slikke et al. [20] validated a three sensor IMU system against a 24-camera optimal motion analysis system across 21 tasks encompassing typical wheelchair basketball tasks. They found an intraclass correlation (ICC) for linear speed to exceed 90% (ICC > 0.90), for rotational speed to exceed 99% (ICC > 0.99) and the instantaneous rotation centre to exceed 90% (ICC > 0.90) all showed very high correlations to the gold standard motion capture. The wheel skid correction algorithm gave a positional error of 0.008 m on average and at high performance, the average difference did not exceed 0.052 m.

## 5. Operational Guidelines

**Figure 4:**
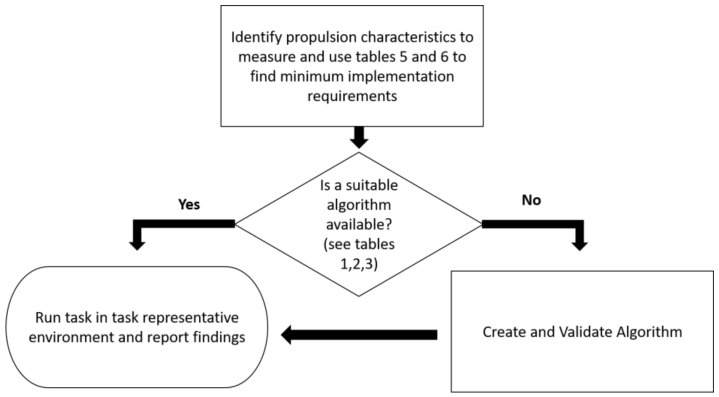
Overall flow chart for wheelchair propulsion measurement.

To reduce the complexity of implementing an IMU for wheelchair propulsion measurement, the flow chart in Figure 4 and Table 6 and Table 7 were developed. These schematics assist the reader to identify the key performance parameters that are desired, determine if a suitable algorithm is available, rate the implementation complexity (simple or advanced), advise on the minimum implementation requirements, and, through the Tables, allow for a quick reference to previous authors’ methodologies.

## 6. Conclusions

From the review, it was evidenced that IMUs are a very useful measurement technology for assessing propulsive characteristics in wheelchair court sports. For any technological implementation to be successful, monitoring it must be user friendly and assessable. The ubiquitous nature of IMUs, the ease of implementation, and the low cost means that the use of this technology has proven its efficacy for these criteria. The review uncovered a wide variety of successful instrumentation implementations in terms of hardware and processing algorithms. These prior successful implementations should serve as a blueprint for future research and development in this area and guide researchers in directing novel research. Authors should also consider collaboration through shared or open-sourced algorithms to reduce the barrier for potential users who do not have the technical abilities to design bespoke software. 

When assessed for validity and reliability, IMUs have shown great promise in providing accurate measurement solutions which should provide coaches and athletes confidence in using this as a measurement tool. As IMUs can be used in real time, it is evident that they have the scope to become a concurrent coaching feedback tool and, with the addition of positional tracking elements, could aid to inform tactical decisions and quantify expert performance.

## Figures and Tables

**Table 1:** Wheelchair performance propulsion element.

Citation	Angular Velocity	Ground Speed	Distance	Trajectory/Position	Turning Radius	Push Cycle Duration	Cadence/Push Frequency	Push force and/or Synchrony	A/C
[16]	√	√	√	√	√	x	x	x	x
[6]	√	√	√	x	√	√	√	x	x
[17]	x	x	x	x	x	x	x	x	√
[18]	x	√	x	x	x	√	√	√	x
[19]	x	√	x	x	x	x	x	x	x
[13]	√	x	x	x	x	x	x	x	x
[14]	√	√	√	√	x	x	x	x	x
[20]	√	√	√	√	√	x	x	x	x
[7]	√	√	√	√	x	x	x	x	x
[21]	√	√	√	√	√	x	x	x	x
[22]	√	√	√	x	x	x	x	x	x
[23]	x	x	x	x	x	x	√	√	x
[24]	√	x	√	√	x	x	x	x	x
[12]	x	x	x	x	x	x	x	x	√
[25]	√	√	√	√	√	x	x	x	x

A/C, activity classification.

**Table 2:** Review of the algorithmic processing performed on the data.

Citation	Frame of Reference Consideration	Wheel Skid Correction	Filtering/Windowing Data	Correction/Offset Factor	Hausdoff Fractal Dimension
[16]	√	x	x	x	x
[6]	√	x	x	x	x
[17]	x	x	√	x	√
[18]	x	x	√	x	x
[19]	x	x	√	x	x
[13]	x	x	√	√	x
[14]	√	√	√	x	x
[20]	√	√	√	x	x
[7]	x	x	√	x	x
[21]	x	x	x	x	x
[22]	x	x	x	√	x
[23]	√	√	√	x	x
[24]	√	x	√	x	x
[12]	x	x	√	x	√
[25]	√	√	√	x	x

**Table 3:** Comparison of the instrumentation used.

Citation	# of IMU	Gyroscope	Accelerometer	Magnetometer	Sampling Frequency	Real-Time	GPS	RF/BT	Wheel	Chair	Wrist
[16]	2	+2000°/s	±3 g	x	30–50 Hz	√	x	20 m’	√	x	x
[6]	2	+1600°/s	x	x	100 Hz	x	x	x	√	x	x
[17]	1	x	±8 g	x	60 Hz	x	x	x	x	√	x
[18]	3	x	±6 g	x	128 Hz	x	x	x	x	√	√
[19]	1	+2000°/s	x	x	50 Hz	x	x	x	√	x	x
[13]	1	+6000°/s	x	x	100 Hz	√	x	30 m’	√	x	x
[14]	3	+2000°/s	±8 g	x	256 Hz	x	x	x	√	√	x
[20]	3	+2000°/s	±8 g	x	256 Hz	x	x	x	√	√	x
[7]	2	+1200°/s	x	x	100 Hz	√	√	x	√	x	x
[21]	1	+1200°/s	x	x	100 Hz	x	x	x	x	√	x
[22]	1	±6000°/s	x	x	64 Hz	√	x	√”	√	x	x
[23]	3	+2000°/s	±8 g	x	200 Hz	x	x	√”	√	√	x
[24]	3	+2000°/s	±16 g	±7 Gauss	250 Hz	x	x	x	√	√	x
[12]	1	x	±8 g	x	60 Hz	x	x	x	x	√	x
[25]	3	±2000°/s	±16 g	±8.1 Gauss	200 Hz	x	x	x	√	x	x

Number (#) of IMU, inertial measurement unit; RF, radiofrequency; BT, Bluetooth; GPS, global positioning system.

**Table 4:** Scientific databases and the associated search parameters used.

Database	Search Terms
Sage Journals	Anywhere (Wheelchair) AND anywhere (sports) AND anywhere (Inertial Sensors)
Proquest	All (Wheelchair) AND all (sports) AND all (Inertial Sensors)
ScienceDirect	“Wheelchair” AND “sports” AND “Inertial Sensors”
Scopus	TITLE-ABS-KEY (“Wheelchair” AND “sports” AND “Inertial Sensors”)
Web of Science	TS = (Wheelchair AND sports AND Inertial Sensors)
Google Scholar	“Wheelchair court sports Inertial Sensors”

**Table 5:** Validation Methods.

Citation	Validation Technologies
[16]	LASER range finder, Ground based markings
[6]	Speed profile from generator
[17]	Validation against recorded video
[18]	High speed video
[13]	Minimax IMU and Ipod Touch IMU
[14]	24 optical motion capture system
[20]	24 optical motion capture system
[21]	Stop watch, set track distance and synced video
[22]	Lathe at set RPM
[24]	LASER range finder, Ground based markings

**Table 6:** Reference to aid the understanding of implementation complexity.

Propulsive Group	Propulsive Element	Implementation Complexity
Wheelchair Kinematics	Acceleration/Velocity/Distance	Simple/Advanced
Athlete Kinematics	Segment Angles/Cadence	Simple/Advanced
Kinetics	Synchronicity/Force/Duration	Simple
Spatiotemporal	Trajectory/Positioning/Turning Radius	Advanced
Sport Specific	Activity Classification	Advanced

**Table 7:** Reference to aid the minimum algorithmic and hardware implementation requirements.

Implementation Complexity	Minimum Hardware Implementation	Minimum Algorithm Implementation
Simple	Single Accelerometer >50 Hz Sampling Frequency Frame Mounting	Threshold based peak detection Noise Reduction filtering
Advanced	≥2 IMUs >50 Hz Wheel/Hub mounting	Advanced signal processing Frame of reference consideration Wheel Skid correction
